# Glass-Based Half-Mode SIW Bandpass Filter with Negative Coupling Structure

**DOI:** 10.3390/mi17020219

**Published:** 2026-02-06

**Authors:** Chen Shi, Wenlei Li, Jihua Zhang, Zhihua Tao, Yong Li, Dongbin Wang, Shuang Li, Ting Liu

**Affiliations:** 1 3D Chips (Guangdong) Technology Co., Ltd., Dongguan 523808, China; fishichen0721@gmail.com (C.S.); rongyi-111@163.com (Y.L.); db.wang@cdmicrotech.com (D.W.); s.li@cdmicrotech.com (S.L.); t.liu@cdmicrotech.com (T.L.); 2 State Key Laboratory of Electronic Thin Films and Integrated Devices, University of Electronic Science and Technology of China, Chengdu 611731, China; tzh3595@uestc.edu.cn

**Keywords:** through glass via, millimeter-wave filter, half-mode substrate integrated waveguide, negative coupling, transmission zeros

## Abstract

This work presents a millimeter-wave half-mode substrate integrated waveguide filter with high selectivity, using through glass via technology. Compared to a traditional printed circuit board, the benefits of high precision and integration afforded by the glass-based process enable the substrate-integrated waveguide to be employed at a higher operating frequency. A novel negative coupling structure is proposed for achieving a quasi-elliptic function response, and its coupling mechanism is investigated to explore the properties of the finite transmission zeros. The proposed coupling slots allow for flexible adjustment of the coupling between the half-mode substrate integrated waveguide cavities from positive to negative by modulating the corresponding geometrical parameters. As a prototype, a glass-based fourth-order bandpass filter is synthesized, simulated, fabricated and measured. Subsequently, good matching is captured, confirming the validity of the topology. The proposed glass-based negative coupling structure is promising for realizing substrate integrated waveguide filters with a quasi-elliptic function response, especially operating at millimeter-wave band.

## 1. Introduction

High bandwidth is recognized as an inevitable trend in the development of high-speed wireless communication systems. Bandpass filters as widely used core components are required to have high selectivity, compact footprint and high bandwidth. In recent years, substrate integrated waveguide (SIW)-based functional components have attracted a great deal of attention. SIW can offer the benefits of both a rectangular waveguide and microstrip circuit, including low loss, high power-handling capability, and easy integration [1,2,3]. Printed circuit board (PCB)-based SIW filters with operating frequencies below the millimeter band have been exhaustively explored [4,5,6].

However, the conventional PCB process hinders the application of SIW in the millimeter wave band [7,8]. The vias fabricated using PCB are oversized, which makes it challenging to achieve effective tuning of the SIW device. Another concern is reliability, including machining accuracy and withstanding temperature. In contrast, the desired through-holes in the glass can be obtained by ultrafast laser-induced etching technology. By virtue of precision machining capability and excellent microwave performance, the emerging glass-based devices are considered to be one of the most promising technologies for implementing RF-integrated passive devices [9,10]. It also implies that the existing SIW technology combined with the process features of through glass via (TGV) can serve as inspiration for developing innovative millimeter wave filters.

The sharp skirt selectivity is essential for a bandpass filter, because practical systems often require the filter to pass the desired channel while strongly suppressing nearby interference just outside the passband. Recent millimeter-wave bandpass filters have demonstrated that introducing multiple transmission zeros (TZs) through multi-mode resonators and additional coupling paths is an effective way to enhance skirt selectivity and stopband rejection [11]. The cross-coupling topology is a well-established approach to obtain a quasi-elliptic response, where finite TZs are intentionally introduced near the passband edges [12]. Intuitively, these TZs act as “deep notches” in the transmission curve, forcing the response to drop rapidly on both sides of the passband and thereby producing much steeper skirts than a conventional Chebyshev response. Such pole–zero placement and its relation to cross-coupling can be systematically described and synthesized using coupling-matrix theory for coupled-resonator filters [13]. These TZs originate from destructive interference between the main signal path and an additional cross-coupling path; introducing a negative coupling effectively provides the required opposite coupling sign (phase inversion), making it an efficient way to generate and tune finite TZs on both sides of the passband [13]. Negative coupling is crucial in generating finite TZs on both sides of the passband [14,15], but the negative-coupling structures for SIW-based devices in millimeter-wave bands are rarely researched and reported. Therefore, developing a negative coupling structure for glass-based millimeter wave filters is critical and pressing work. In addition, SIW-based configurations still suffer from excessive occupied areas. Fortunately, the miniaturization approach for PCB-based SIWs can be extended to glass-based devices, such as half-mode substrate integrated waveguide (HMSIW) [16], SW-SIW [17], FSIW [18] and multilayer SIW [19].

Motivated by recent progress in glass-based TGV-enabled SIW filters, demonstrating quasi-elliptic responses at millimeter-wave frequencies, this work proposes a compact, geometry-tunable negative-coupling structure for glass-based HMSIW cavities and demonstrates a fourth-order Ka-band bandpass filter with a quasi-elliptic response [20,21]. HMSIW cavities are employed to decrease the occupied area. Furthermore, the coupling properties between the HMSIW cavities are exhaustively investigated to reveal the TZ’s characteristics. Subsequently, a fourth-order HMSIW filter with a negative coupling structure is circuit synthesized and simulated. The filter prototype is fabricated and measured using the glass-based vertical interconnect technology and semiconductor thin-film planar process. The experimental results confirm the feasibility of the proposed glass-based negative coupling structure.

## 2. Filter Design and Analysis

### 2.1. Filter Configuration

**Figure 1:**
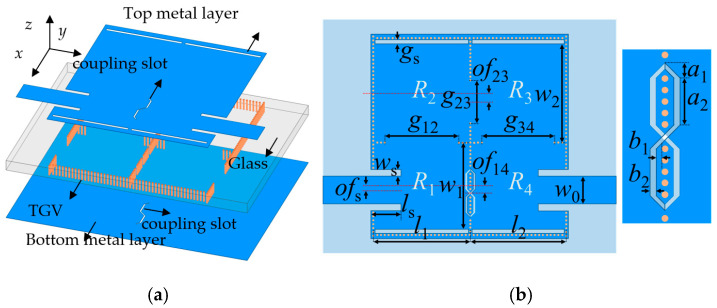
Schematic view of the proposed glass-based filter. (**a**) Exploded 3D view. (**b**) Top view.

Figure 1 illustrates the configuration of the glass-based filter, which is composed of two metal layers with etched slots and a glass substrate with metallized vias. The proposed fourth-order filter is composed of four half-mode SIW resonators. The half-mode SIW is derived by bisecting a conventional SIW along its equivalent magnetic wall. To minimize leakage loss, a row of metallized TGVs is placed at a distance $g_{s}$ from the open edge of the half-mode SIW resonator. Consequently, a gap is inherently present in every half-mode SIW resonator. The proposed negative coupling structure shown in Figure 1b consists of a pair of mirror-symmetric coupling slots, which surround two columns of metallized TGVs. Both electric and magnetic coupling can be induced between the resonators $R_{1}$ and $R_{4}$. The diameter and height of the TGV are set to 60 μm and 400 μm, respectively. The pitch between adjacent metallized vias is fixed to 120 μm. In addition, a low-loss fused silica with a dielectric constant of 3.8 is selected as the glass substrate.

### 2.2. Coupling Characteristics

The type of coupling is determined based on the direction of the magnetic field in the two half-mode resonators at the low mode and high mode. When two identical resonators are coupled, they produce two split resonant modes (dual peaks: the low mode and the high mode, typically corresponding to the odd and even modes). When the coupling between the two resonators is primarily electrical (capacitive coupling), the lower-frequency coupled mode (odd mode) exhibits opposite magnetic field directions, while the higher-frequency coupled mode (even mode) exhibits the same magnetic field direction. Similarly, under magnetic coupling, the low-frequency mode (even mode) corresponds to in-phase magnetic fields, and the high-frequency mode (odd mode) corresponds to out-of-phase magnetic fields.

**Figure 2:**
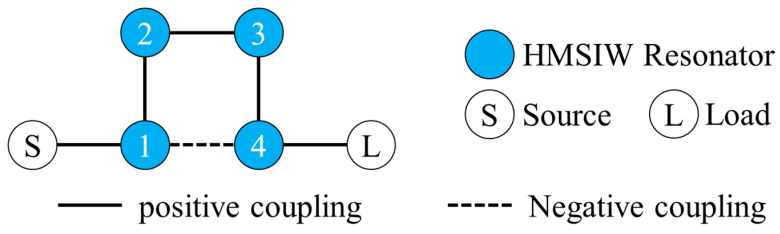
Coupling topology of the proposed glass-based filter.

The coupling topology of the fourth-order HMSIW filter is presented in Figure 2. The coupling characteristics of $R_{1}$ and $R_{4}$ depend on the physical dimension and location of the coupling slots. Actually, the length $a_{2}$ of the coupling slot is determined by the number of TGVs it surrounds. Based on the simulation results from Advanced Design System software 2020 version, the coupling matrix of the proposed filter is shown below:

$$M=\left[\begin{array}{cccc} -0.0288 & 0.9579 & 0 & -0.2\\ 0.9579 & -0.0298 & 0.8239 & 0\\ 0 & 0.8239 & -0.0298 & 0.9579\\ -0.2 & 0.9579 & 0 & -0.0288 \end{array}\right]$$

**Figure 3:**
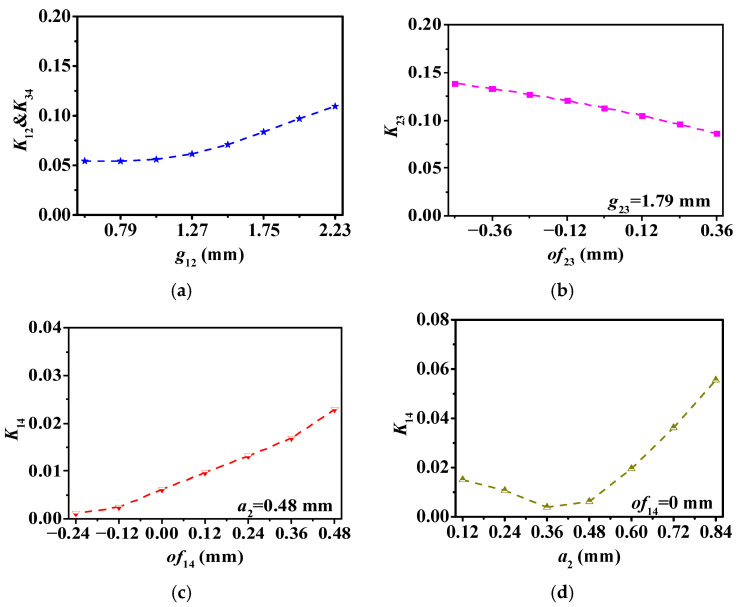
Extracted coupling coefficients. (**a**) $K_{12}$ and $K_{34}$ between $R_{1}$ ($R_{3}$) and $R_{2}$ ($R_{4}$). (**b**) $K_{23}$ between $R_{2}$ and $R_{3}$; (**c**,**d**) $K_{14}$ between $R_{1}$ and $R_{4}$.

As anticipated, a positive dependency correlation is found between $K_{12}$ and $K_{34}$ and $g_{12}$. In addition, $K_{23}$ gradually increases as the iris approaches the equivalent magnetic wall. As observed in Figure 3c, the coupling strength enhances with the increasing ${of}_{14}$. This phenomenon is attributed to the electric field distribution within the HMSIW cavity. The strength of the electric coupling increases as $a_{2}$ becomes larger. In Figure 3d, it is worth mentioning that the coupling coefficient $K_{14}$ demonstrates a trend of initially decreasing and then increasing with the rise of $a_{2}$.

**Figure 4:**
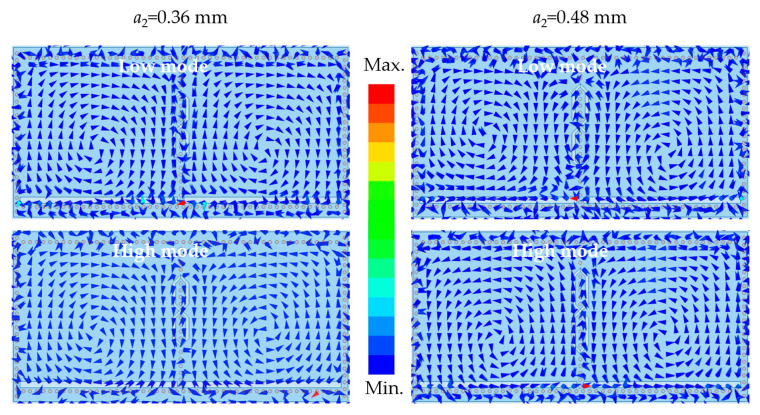
Magnetic field distribution of the $R_{1}$ and $R_{4}$, adjacent to the negative coupling structure.

To further analyze the underlying mechanism, the magnetic field distributions of the $R_{1}$ and $R_{4}$, adjacent to the negative coupling structure, are plotted in Figure 4. When $a_{2}$ = 0.36 mm, $R_{1}$ and $R_{4}$ have the same magnetic field direction at the lower mode, while the opposite magnetic field direction is captured at the higher mode. This magnetic field characteristic suggests that the magnetic coupling plays a dominant role, which is consistent with the results in [11]. The opposite case of magnetic field variation is found when $a_{2}$ = 0.48 mm, which implies that negative coupling occurs between $R_{1}$ and $R_{4}$. The proposed negative coupling structure can fulfill the capability of flexibly regulating the transition from positive to negative coupling between the HMSIW cavities.

**Figure 5:**
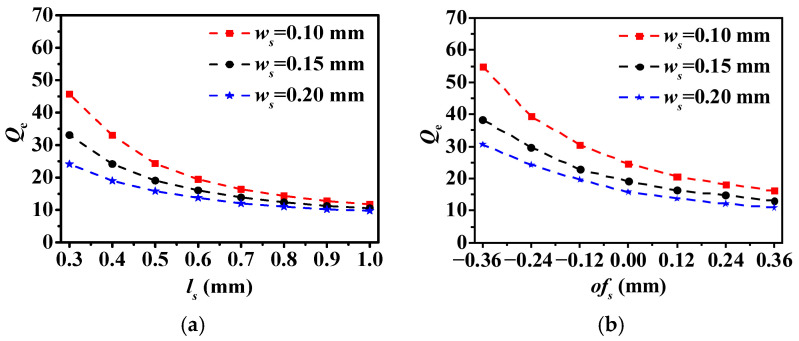
Extracted external $Q_{e}$ versus (**a**) $l_{s}$ and (**b**) ${of}_{s}$ with ws as a variable.

Figure 5 illustrates the extracted external $Q_{e}$, which can be acquired as:

(1) $$Q_{e}=\frac{W_{c}\cdot \tau S_{11}(W_{c})}{4}$$

where the τ$S_{11}$($W_{c}$) denotes the value of the group delay at the resonant frequency $W_{c}$. The core significance of this formula lies in its direct linkage of the abstract external coupling strength $Q_{e}$ with a simulatable or measurable physical quantity. In fact, the group delay at the resonant point directly reflects the duration for which energy is “trapped” within the resonator and is proportional to $Q_{e}$. As $l_{s}$ or $w_{s}$ increases, more energy is coupled into the HMSIW cavity, resulting in a gradual decrease in $Q_{e}$. The same trend can be obtained with increasing ${of}_{s}$.

### 2.3. TZs Characteristics

**Figure 6:**
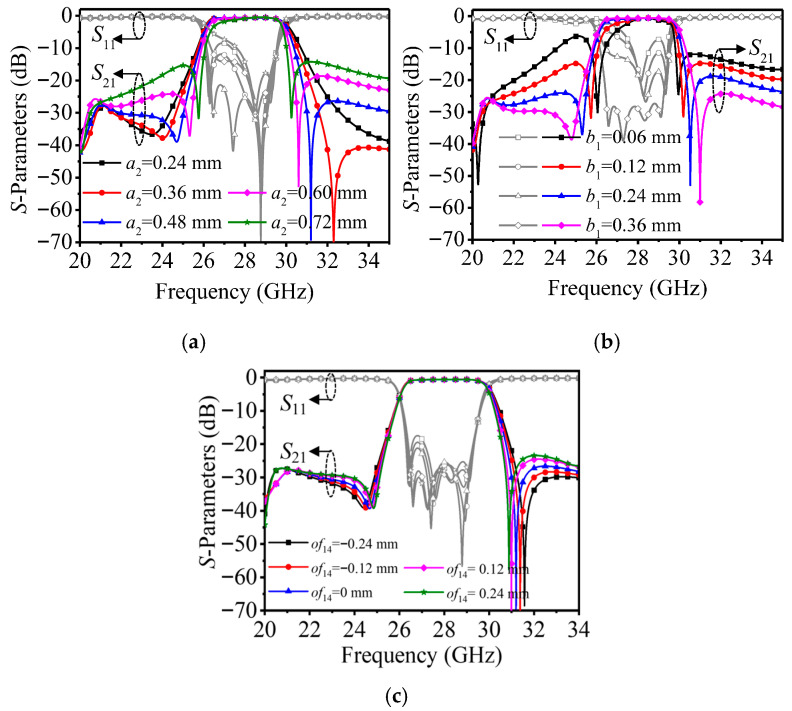
Simulated responses of the fourth-order filter versus the frequency with (**a**) $a_{2}$, (**b**) $b_{1}$ and (**c**) ${of}_{14}$ as parameters varying the negative coupling position offset.

To further analyze the selectivity, the simulated S-parameters under different negative coupling parameters are presented in Figure 6. It can be observed that two TZs are introduced on both sides of the passband, forming a quasi-elliptic filtering response. As shown in Figure 6a, with the increase of $a_{2}$, the TZs gradually move toward the center frequency. Combined with the coupling analysis results, this phenomenon indicates that a stronger negative cross-coupling path requires more energy in the main signal path to achieve cancelation and, thus, the attenuation poles shift closer to the passband.

Similarly, Figure 6b shows that increasing $b_{1}$ strengthens the electric field interaction around the negative coupling region, leading to enhanced cross-coupling and a further movement of the TZs toward the passband edges. Moreover, Figure 6c demonstrates the effect of the negative coupling position offset ${of}_{14}$. With a larger ${of}_{14}$, the electric coupling becomes stronger, and the TZs shift toward the center frequency accordingly. It is worth noting that excessively strong negative coupling may degrade the far-out stopband suppression; therefore, the negative coupling parameters should be carefully optimized to balance skirt selectivity and out-of-band rejection. Overall, the proposed negative coupling structure provides a flexible and practical approach to tune the TZ locations in a glass-based millimeter-wave HMSIW filter.

## 3. Fabrication and Measurement

**Figure 7:**
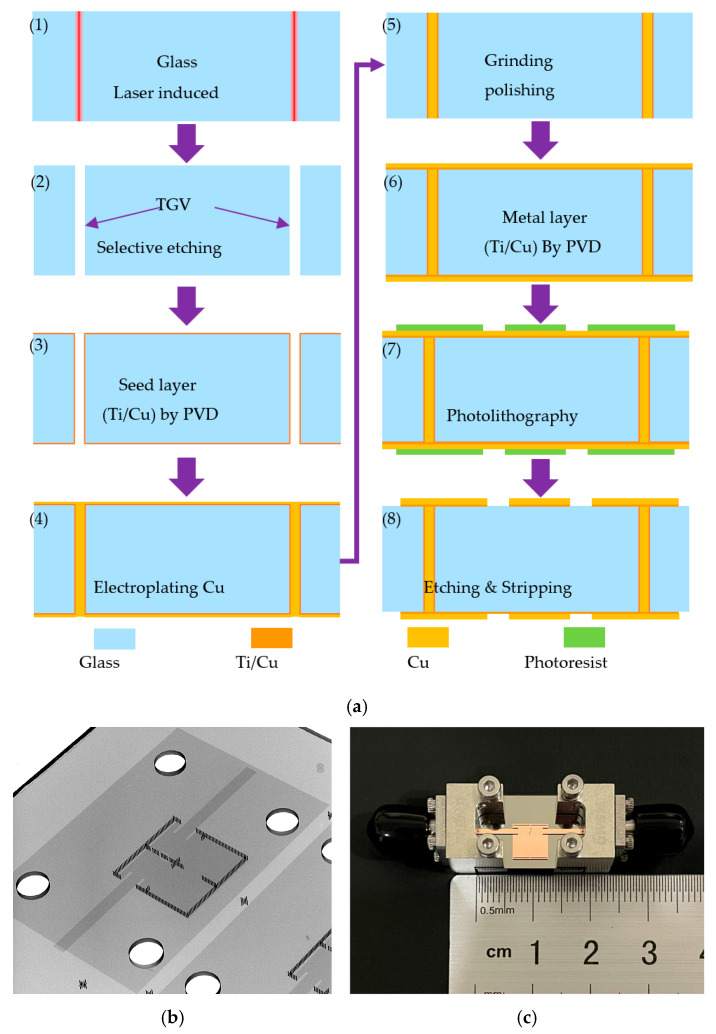
The fabricated filter. (**a**) Schematic diagram of the process flow. (**b**) X-ray detection. (**c**) Physical picture.

As an experimental prototype, a glass-based fourth-order HMSIW bandpass filter is fabricated and experimentally characterized to validate the proposed negative-coupling topology and the corresponding quasi-elliptic response at the Ka-band (around 28 GHz). As illustrated in Figure 7a, the proposed device is demonstrated by integrating vertical TGV interconnect processing with planar semiconductor thin-film fabrication.

First, a 2 inch quartz glass wafer substrate is prepared and its surface is carefully cleaned to remove contaminants, especially particles and organic residues. This pretreatment is essential because surface contamination may disturb the laser-induced modification and the subsequent selective etching process, resulting in smaller vias or even missing vias.

Next, the TGVs are formed using laser-induced selective etching. In this step, the Femtosecond pulse laser irradiation modifies the glass structure in the predefined via locations, and the modified regions are then selectively etched to create through-glass vias with well-controlled profiles. Compared with conventional mechanical drilling, this approach enables higher dimensional accuracy and better consistency, which is critical for constructing stable SIW sidewalls at millimeter-wave frequencies. For instance, deviations in via diameter or sidewall roughness may cause the performance of manufactured filters to deviate from expectations. After via formation, the substrate is thoroughly rinsed and dried to remove etching by-products, followed by the deposition of a Ti/Cu seed layer by physical vapor deposition (PVD). The Ti layer serves as an adhesion layer to enhance metal-to-glass bonding, while the Cu layer provides an initial conductive path for subsequent electroplating. In practice, the continuity and coverage of the seed layer, particularly along the via sidewalls, must be carefully ensured; otherwise, insufficient coverage of the seed layer acting as an electrode may result in uneven current distribution during copper deposition, leading to discontinuous metallization inside the vias.

Copper electroplating is then performed to metallize the TGVs and to increase the conductor thickness. This step plays a crucial role in reducing conductor loss in the Ka-band and enhancing the reliability of vertical interconnects. To achieve void-free and uniform plating, the electroplating conditions need to be properly optimized such that the copper deposition can conformally cover the via sidewalls without forming seam voids or trapped cavities. During plating, the concentrations of major additives (brightener, suppressor, and leveler) are maintained within the target window to prevent via-mouth closure and to promote uniform copper growth along the via sidewalls and interior. In addition, process parameters such as electrolyte temperature, wafer agitation/swinging, and spray-assisted solution circulation are carefully controlled to stabilize the transport of ions and additives throughout the long-duration plating. Specifically, sufficient agitation and spray-driven flow help to reduce concentration polarization and enhance ion/additive replenishment inside the vias, thereby mitigating local depletion and promoting more uniform deposition along the via depth. This control enables consistent void-free metallization and improves the wafer-level yield and repeatability.

Subsequently, surface grinding and polishing are performed to remove the uneven surface copper that grows during the TGV metallization process. Moreover, this process helps us to achieve a uniform substrate thickness and a smoother surface, which improves lithography alignment accuracy and reduces the variability in electromagnetic performance. Finally, planar thin-film processes are employed to define the resonator cavities, coupling slots, and feeding structures on the metal layers. Photolithography is first used to transfer the designed patterns onto the photoresist, followed by metal etching to obtain the desired conductor geometry. After etching, the remaining photoresist is stripped to complete the pattern definition. It should be noted that the dimensional accuracy of the coupling apertures and slot structures directly affects the coupling strength and the TZs locations. Therefore, maintaining tight control of lithography and etching steps is essential for achieving the designed quasi-elliptic response.

As shown in Figure 7c, the fabricated filter occupies a compact circuit footprint of approximately 1.1$\lambda_{g}$ × 1.12$\lambda_{g}$, demonstrating that the proposed HMSIW configuration and the integrated negative coupling structure enable high selectivity while maintaining a small electrical size that is suitable for glass-based mmWave integration.

**Figure 8:**
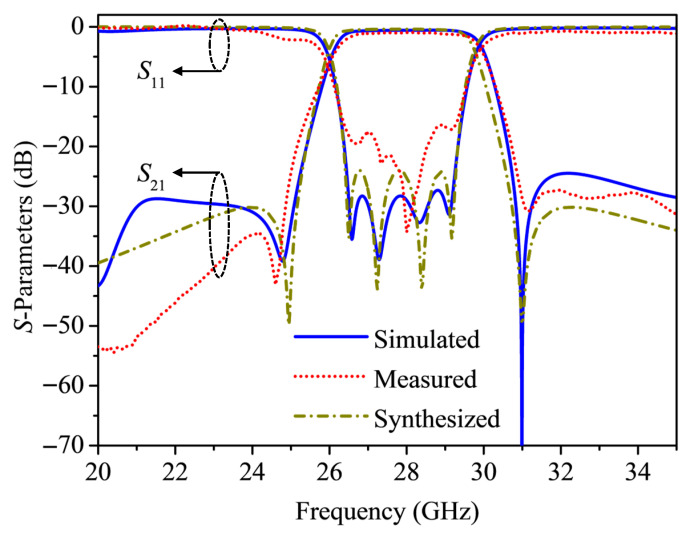
Simulated, synthesized and measured responses versus the frequency. Geometric parameters (mm): $w_{0}$ = 0.88, $w_{1}$ = 2.43, $w_{2}$ = 3.23, $w_{s}$ = 0.17, $l_{1}$ = $l_{2}$ = 2.95, $l_{s}$ = 0.92, $g_{12}$ = $g_{34}$ = 0.12, $g_{23}$ = 0.16, $g_{s}$ = 0.11, ${of}_{23}$ = 0.24, ${of}_{14}$ = 0.12, ${of}_{s}$ = 0.2, $a_{1}$ = 0.15, $a_{2}$ = 0.48, $b_{1}$ = 0.09 and $b_{2}$ = 0.06.

Figure 8 presents the circuit’s synthesized, simulated and measured S-parameters. A little deviation can be observed, which is mainly caused by the SMA connection and the tolerances of the manufacturing process. The measured glass-based filter operates at 28.06 GHz with a 3 dB fractional bandwidth of 12.72%. A return loss better than 16.43 dB and an insertion loss of 0.95 dB can be achieved. Furthermore, the TZs distributed at 24.6 and 31.1 GHz confirm that the proposed negative coupling topology can be used to achieve a millimeter-wave filter with quasi-elliptic function response.

Subsequently, a comparison of the proposed glass-based filter with representative SIW-based designs is summarized in Table 1. To the best of the authors’ knowledge, glass-based SIW (or HMSIW) filters incorporating a compact negative coupling structure for controllable cross-coupling and TZs generation have rarely been reported in the literature. Although TZs close to the passband can be achieved in conventional PCB-based SIW filters using cross-coupling techniques [22,23,24], the millimeter-wave performance is often limited by PCB manufacturing uncertainties. Typical issues include via-hole oversizing and tolerance, limited patterning accuracy for narrow coupling apertures, imperfect layer registration, and increased conductor loss due to copper surface roughness, which may collectively cause deviations in center frequency, insertion loss, and stopband rejection compared with ideal simulations. In addition, a manufacture-friendly folded SIW filter has been reported in [18], but further improvement in selectivity is still required. High out-of-band suppression can be achieved using multilayer LTCC technology [8]; however, the complex stacked process typically increases the development cycle and fabrication cost.

In contrast, the proposed glass-based design provides several advantages for mmWave integration. The glass/TGV platform offers improved dimensional controllability and fabrication repeatability, enabling more stable performance at Ka-band frequencies. Moreover, the proposed negative coupling structure provides a compact and geometry-tunable solution to implement cross-coupling in HMSIW cavities, which enables multiple controllable TZs and enhances skirt selectivity without requiring multilayer stacking or significant footprint expansion. Finally, by adjusting the negative coupling geometry and the feed parameters, the TZs locations and external $Q_{e}$ can be co-optimized to meet various system requirements such as wider fractional bandwidth or steeper rejection. Based on recent semi-symbolic analysis, the poles and zeros of a transfer function can be obtained by casting the circuit/network equations into a matrix form and solving the associated eigenvalue problem, which provides a systematic basis for interpreting TZs as transfer-function zeros [25]. Therefore, the proposed filter topology can serve as a practical building block for highly integrated mmWave front-end modules and advanced packaging-oriented passive networks.

## 4. Conclusions

This work has presented a glass-based fourth-order HMSIW bandpass filter incorporating a compact negative coupling structure to achieve a quasi-elliptic filtering response at the Ka-band. By exploiting the geometry-dependent mixed-coupling behavior, the proposed structure enables a controllable coupling-sign transition and provides an effective cross-coupling path to generate multiple TZs, thereby enhancing skirt selectivity. A prototype has been fabricated using TGV-based glass processing, and the measured results show good agreement with synthesis and full-wave simulations, validating the feasibility and robustness of the proposed approach. Benefiting from the high precision and scalability of the glass/TGV platform, the proposed filter topology is promising for compact and high-selectivity mmWave front-end integration, and it can be further extended toward higher-frequency bandwidth-demanding applications.

## Figures and Tables

**Table 1:** Comparison between the proposed filters with other works.

Ref.	$f_{0}$ * (GHz)	Order/Layer	Fractional Bandwidth (%)	Insertion Loss (dB)	Transmission Zeros	Size	Complexity of Design and Process	Implementation Technology
[8]	28	4/11	20	0.8	2	$1.11\lambda_{g}\;*\;\times \;0.58\lambda_{g}$	complex	LTCC
[18]	25.88	4/2	4.48	4.36	0	$1.57\lambda_{g}\;\times \;1.03\lambda_{g}$	simple	PCB
[22]	10	4/1	3.3	1.55	2	$1.3\lambda_{g}\;\times \;1.53\lambda_{g}$	simple	PCB
[23]	15.3	2/2	3	2.4	2	$1.18\lambda_{g}\;\times \;1.18\lambda_{g}$	moderate	PCB
[24]	10.1	4/1	3.98	1.52	1	$1.31\lambda_{g}\;\times \;1.51\lambda_{g}$	moderate	PCB
This work	28.06	4/1	12.72	0.95	2	$1.1\lambda_{g}\;\times \;1.12\lambda_{g}$	simple	TGV

* $f_{0}$: the center frequency, $\lambda_{g}$: the guided wavelength at $f_{0}$.

## Data Availability

The original contributions presented in this study are included in the article. Further inquiries can be directed to the corresponding authors.
